# Using animated vehicles with real emotional faces to improve emotion recognition in Chinese children with autism spectrum disorder

**DOI:** 10.1371/journal.pone.0200375

**Published:** 2018-07-19

**Authors:** Yuhong Yan, Chuanshi Liu, Lin Ye, Yangyang Liu

**Affiliations:** 1 Department of Psychology, Nanjing Normal University, Nanjing, China; 2 School of Media and Design, Shanghai Jiaotong University, Shanghai, China; 3 Department of Psychology, Nanjing University, Nanjing, China; 4 School of Education, Tianjin University, Tianjin, China; Univdersity Hospital of TübingenUniversitatsklinikum Tubingen, GERMANY

## Abstract

The objective of the present study was to conduct an intervention study which aimed to improve emotion recognition for Chinese children with ASD by using animated vehicles with real emotional faces. A total of 21 children participated in the current study; participants consisted of 14 children (2 girls) with a formal diagnosis of ASD and 7 typically developing children. Participants were measured on emotional vocabulary and situation-facial expression matching before and after the intervention. Results indicated that the intervention significantly improved ASD children’s emotion recognition compared to their pre-intervention scores. Our findings suggest that this emotional recognition intervention using animated vehicles (i.e. The Transporters) is an effective early intervention for Chinese children with ASD.

## Introduction

In previous research, several researchers have suggested that an early intervention starting during the toddler or preschool years can modify the impairments that influence the developmental trajectories of children with autism spectrum disorder (ASD) [1]. A number of studies revealed the interventions that were useful in improving ASD children’s social-communication functioning [2,3]. Children with ASD usually demonstrate difficulties in understanding the emotional and mental states of other ones [4]. Recently, based on the Empathizing–Systemizing (E–S) theory [5], some researchers have created a series of animated vehicles with real emotional faces (i.e. The Transporters) to improve children’s abilities on emotion recognition [4]. Their findings indicate this method can enhance the abilities of emotion recognition in children with ASD [4]. Although nearly 20% of the world population is Chinese, studies on ASD are largely lacking in China [6]. Intervention studies have rarely been conducted among Chinese children with ASD. Therefore, the present study sought to conduct an intervention study which aimed to examine the effect of the transporters animated vehicles with real emotional faces on Chinese children with ASD in their emotion recognition. We expect that Chinese children with ASD, who receive the intervention, will demonstrate improvements in their emotion recognition.

## Method

### Participants and procedures

The study was approved by the institutional review board of Nanjing normal university, and informed consent was obtained by the participants’ parents. All procedures performed in studies involving human participants were in accordance with the ethical standards of the institutional and/or national research committee and with the 1964 Helsinki declaration and its later amendments or comparable ethical standards. The sample consisted of 21 Chinese children. Fourteen children (2 girls) had a formal diagnosis of ASD from a professional hospital, and 7 (1 girl) children were typically developing (TD). Their mean age was 67.10 months (SD = 10.95 months). The participants with ASD were from a non-profit center serving the city of Nanjing and surrounding rural areas, China. These participants were equally divided into two groups (an ASD intervention group and an ASD control group). The two groups were matched for sex, age, verbal ability, and IQ (using the Wechsler Preschool and Primary Scales of Intelligence). The TD children were from a local school in the same city. We first measured all the participants’ abilities on emotional vocabulary and situation-facial expression matching before the intervention (Time 1). In the next 6 weeks, we used the intervention materials to train these children. The intervention was completed in the non-profit center by a postgraduate student of psychology. The participants in the ASD intervention group (n = 7) were taught by a postgraduate student each day, five days a week, for 40 minutes. After the 6-week intervention, we tested all the children’s skills again (Time 2).

### Measures

#### Emotional vocabulary

Similar to the task used in Golan el al [4]’s study to measure emotional vocabulary, the task used in the present study required the participants to define 15 emotion words, which are key emotions (i.e. happy, sad, angry, afraid, disgusted, surprised, excited, tired, unfriendly, kind, sorry, proud, jealous, joking and ashamed) in the intervention materials and give examples of situations that evoke them [4]. For example, the researcher asked the participants “how do you feel when you get your favorite sugar?" and showed them a picture with an emotion which was selected from the intervention materials. Participants’ responses were recorded. One correct response received one point and a total score of this task is 15 points. Considering the cultural differences and word-using habits, our study didn’t use the emotional vocabulary word ‘worried’ like Golan et al. [4] did in their study.

#### Situation-facial expression matching task

This task has five items that covered five basic emotions (i.e. happiness, anger, sorrow, fear, and astonishment). The five basic emotions were selected according to the frequencies of emotional words used by children (age 4 to 8) in China. Each item has a picture depicting a scene with a short description from “The Transporters” video. The researcher read out the description of the scene and then asked participants to choose the correct corresponding facial expression from a set of three different facial expressions (the target and two foils) that described the feeling of the character in the scene. Correctly describing the feeling earned one point and correctly selecting the corresponding expression earned one point for a maximum score of two points per question.

#### Interventional materials

**Fig 1:**
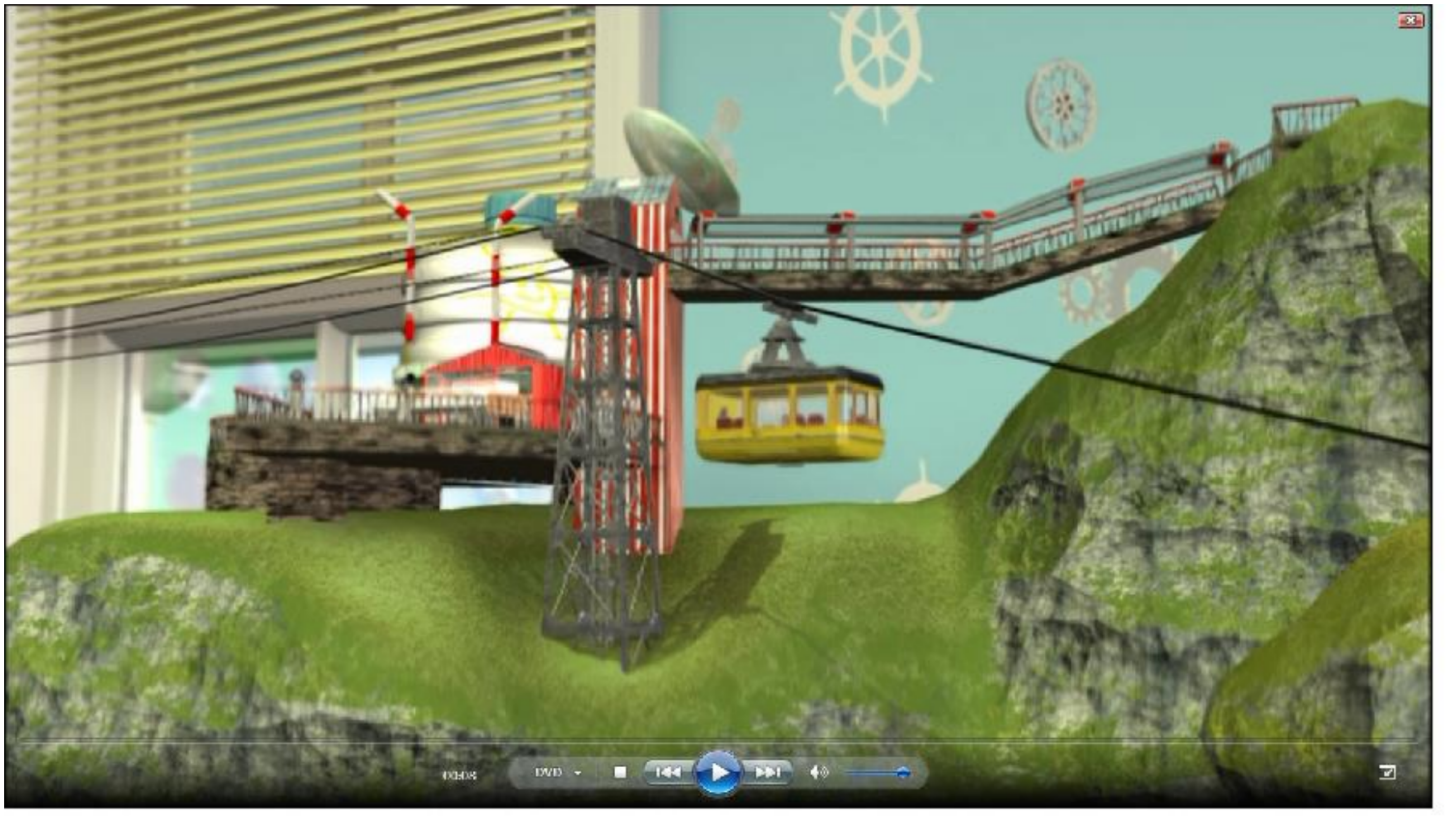
An example of interventional materials.

The Transporters DVD (http://www.thetransporters.com/) was used as the basis for the intervention materials (an example is illustrated in Fig 1). The materials were translated into Chinese, some of the original materials were adapted according to Chinese culture and language. The aim of the intervention materials is to improve ASD children’s abilities on emotion recognition.

## Results

**Fig 2:**
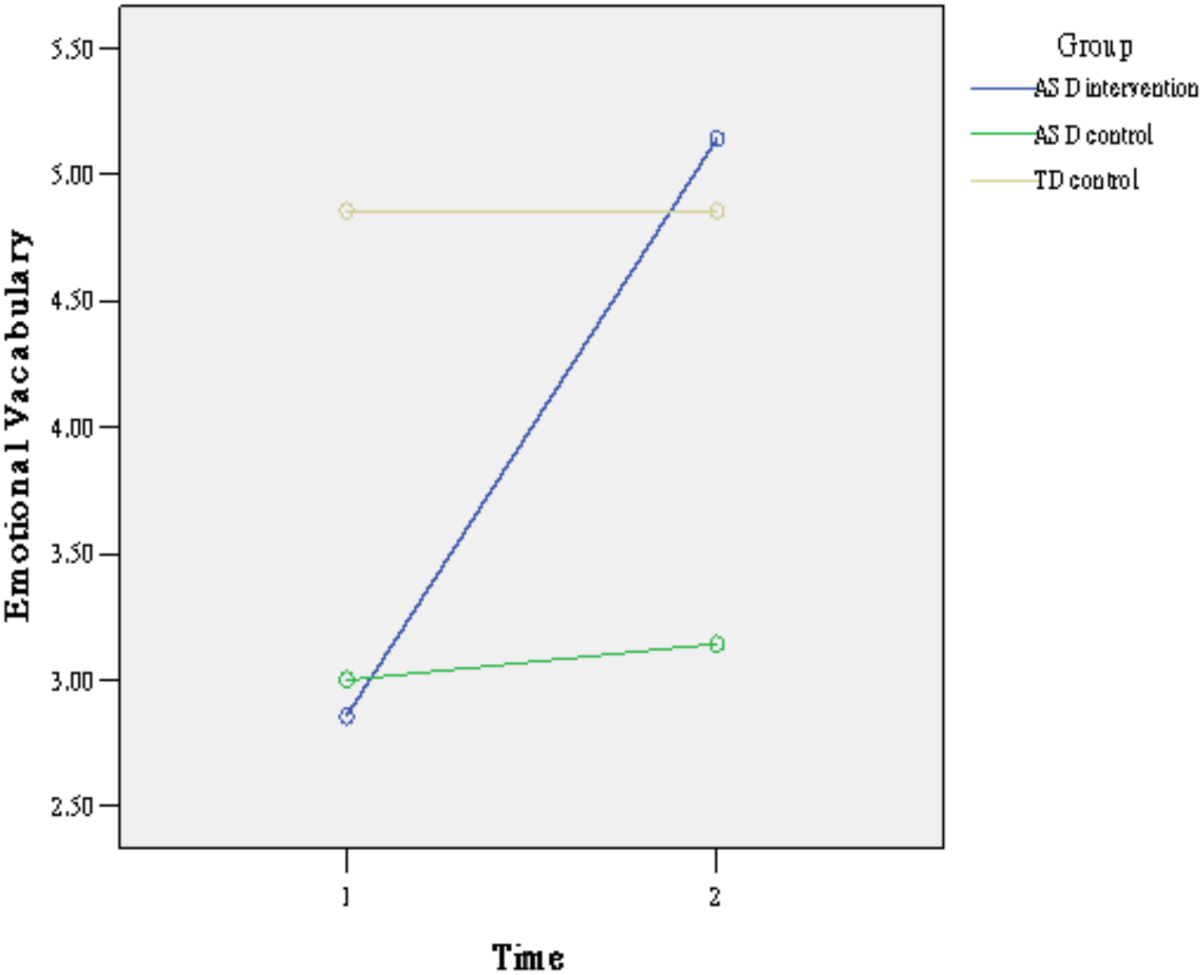
The effect of the intervention on emotional vocabulary by group.

**Fig 3:**
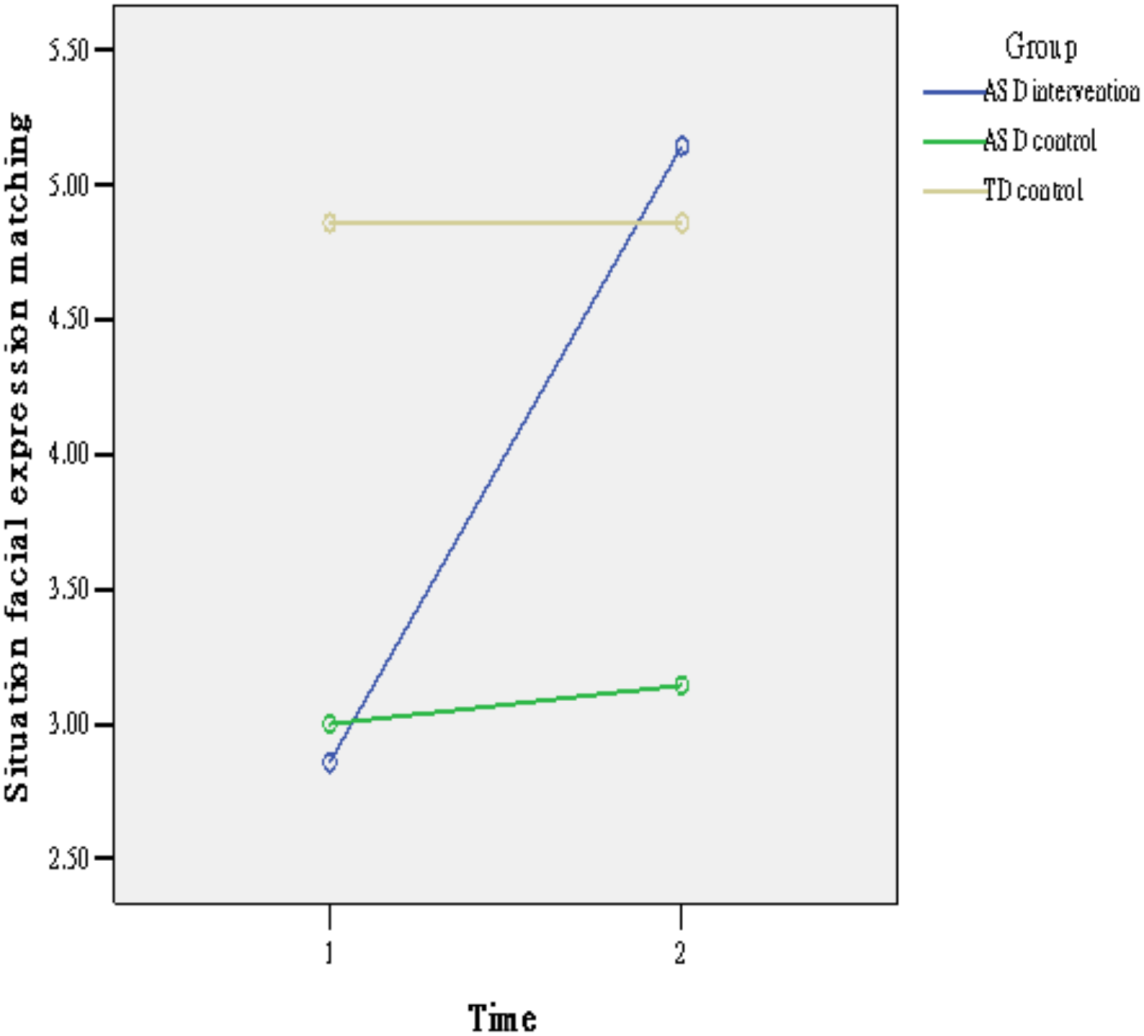
The effect of the intervention on situation-facial expression matching by group.

Means and standard deviations (SD) of children’s abilities on emotional vocabulary and situation-facial expression matching at two time points are shown in Table 1 separated by group. Repeated ANOVAs were used to examine the differences of children’s abilities before and after the intervention. As shown in Table 2, the main effects of time and the interaction of time by group were significant on emotional vocabulary. Simple main effect analysis (Fig 2) revealed that the ASD intervention group (t[6] = 5.28, p <. 01) and ASD control group (t[6] = 2.82, p < .05) improved significantly between the two time points, while the typical control group did not (t[6] = 1.00, p > .05). For the situation-facial expression matching task, as shown in Table 2, the results indicated that both the main effect of time and the interaction of group × time were significant. Simple main effect analysis (Fig 3) showed that only the ASD intervention group (t_[6]_ = 3.20, p < .05) significantly improved between the two time points.

**Table 1:** Means and SDs on emotional vocabulary and situation-facial expression matching of the three groups.

		Emotional Vocabulary		Situation-Facial Expression Matching	
		Mean	SD	Mean	SD
ASD-intervention (N = 7)	Time1	2.29	2.50	4.14	3.08
	Time2	5.43	3.74	8.00	2.52
ASD-control (N = 7)	Time1	2.43	2.51	3.28	3.50
	Time2	3.00	2.52	4.00	3.70
Typical-control(N = 7)	Time1	5.85	1.95	5.71	2.06
	Time2	4.86	1.46	4.86	1.46

**Table 2:** Repeated measures ANOVA for emotional vocabulary and situation-facial expression matching.

	Group		Time		Group×Time	
	F_(2,18)_	η^2^	F_(2,18)_	η^2^	F_(2,18)_	η^2^
Emotional Vocabulary	2.57	0.22	30.74**	0.63	21.59**	0.71
Situation-Facial Expression Matching	0.90	0.09	9.42**	0.34	7.86**	0.47

** p < .01,

*** p < .001

## Discussion

The present study contributes to the literature in a number of important ways. First, to our knowledge, it is the first study that explores a way to improve Chinese ASD children’s emotion recognition. In previous research, Golan et al.’s [4] study found that “The Transporters animated series” was significantly beneficial to emotion comprehension and recognition skills for Western children. Comparing Chinese ASD children’s abilities at the beginning and end of the treatment, meaningful increases of abilities were found in this study. Our findings suggest that the DVD is also an effective early intervention for Chinese children. Second, the previous research focused on children with HFA/AS [4]. The present study found that “The Transporters animated series” can be useful in helping children with ASD as well. Third, in the previous Western study [4], the intervention was conducted through parental tutoring. In this study, a trained post-graduate student completed the intervention by teaching the children, which can increase the accuracy of the present findings.

In the present study, there are some limitations that could be improved in future research. First, the intervention only lasted for 6 weeks, which is brief for measuring change. A longer intervention can be used to examine whether there are greater improvements over a longer period. Furthermore, there were only two girls with ASD who participated in the present study. In further research, more girls could be recruited to examine the gender differences in the effect of the intervention. In addition, our study does not have a placebo group; such a group could be used in further research.

## Supporting information

S1 Dataset CN1 dataset. (SAV)
